# Making Room for Mental Health in the Medical Home

**Published:** 2010-10-15

**Authors:** Michael F. Hogan, Lloyd I. Sederer, Thomas E. Smith, Ilana R. Nossel

**Affiliations:** New York State Office of Mental Health, New York, New York; New York State Office of Mental Health; New York State Office of Mental Health, New York, New York. Dr Smith is also affiliated with Columbia University College of Physicians and Surgeons, New York, New York; New York State Office of Mental Health, New York, New York. Dr Nossel is also affiliated with Columbia University College of Physicians and Surgeons, New York, New York

## Abstract

Discussions of health care reform emphasize the need for coordinated care, and evidence supports the effectiveness of medical home and integrated delivery system models. However, mental health often is left out of the discussion. Early intervention approaches for children and adolescents in primary care are important given the increased rates of detection of mental illness in youth. Most adults also receive treatment for mental illness from nonspecialists, underscoring the role for mental health in medical home models. Flexible models for coordinated care are needed for people with serious mental illness, who have high rates of comorbid medical problems. Programs implemented in the New York State public mental health system are examples of efforts to better coordinate medical and mental health services.

## Introduction

*Home is the place where, when you have to go there, they have to take you in.*

Robert Frost, *The Death of the Hired Man*

The debate on health care reform is focused on expanding insurance coverage, but reform ultimately turns on improved care. An improved health care system must emphasize primary and preventive care, improving health through earlier and less costly care, while ensuring quality care when serious or complex illness emerges. Options to deliver integrated care include the *medical home*, large integrated care systems, such as the Mayo Clinic or Kaiser Permanente (1), and smaller integrated community health care systems (2-4). Each of these approaches focuses on planned, integrated, and coordinated medical services — largely provided by teams in primary care settings.

As this country considers its medical future, it is time to integrate mental health care with general medical care. We review key elements of medical home and coordinated care models and describe how these approaches enhance quality and outcomes for 1) children and adolescents, for whom early detection and treatment of mental illness is critical; 2) the general adult population, which receives the bulk of its mental health care in medical settings; and 3) people with serious mental illnesses, who increasingly receive both their mental and primary care in mental health settings.

## Key Elements of the Medical Home and Coordinated Care Models

The medical home model originated several decades ago as an approach to coordinating services for children with special health care needs (2). The model has garnered attention in recent health care reform discussions as a potential solution to escalating costs and poor access to primary care and preventive services (5). Principles of the medical home include enhanced access to care, an ongoing relationship with a personal physician, orientation to the whole person, a team approach to care, coordinated or integrated care, and a commitment to quality and safety (6).

A review of 30 studies of the medical home model in pediatric care indicated that adoption of medical home principles is associated with better health status, better access to care, and improved family functioning for children and adolescents (7). Studies of adults also show that care coordination programs have enhanced quality and greater consumer satisfaction compared with traditional fee-for-service reimbursement approaches (8). Hawaii and North Carolina have taken the lead in efforts to implement statewide public health system reforms (9,10). These states demonstrated that medical home or integrated care approaches are associated with improved access to care, greater use of preventive services, and diminished rates of crisis intervention services and emergency department use (9,10).

To be viable, medical home and integrated care models need to demonstrate cost-effectiveness. The most compelling data come from North Carolina's Community Care model, which estimated Medicaid savings of $50 million to $260 million per year in 2003 and 2004 following implementation of care networks that incorporate medical home principles (11). Although more research is needed, experts remain optimistic that coordinated care approaches will bring cost savings (12).

A central aspect of the medical home model is *point of care*. In the medical home model, a primary care physician takes responsibility for coordinating services provided by a team of clinicians. For most adults with mental illness, the point of care would be an internist or family practitioner. People with serious mental illness, however, typically receive most of their care from a mental health clinic. For this population, the successful medical home approach requires a more flexible notion of point of care. Psychiatrists treating people with serious mental illness should monitor basic medical conditions and communicate with primary care practitioners, who provide guidance and specific treatment recommendations (13).

## Integration of Mental Health Into Primary Care

Integration of mental health into primary care is critical given the high prevalence of mental illness (14), the interconnectedness of mental and medical illness (15), and the limited availability of specialized mental health services (16). The prevalence of mental illness in the US population is estimated to be 26% (14). Most people with a mental illness do not receive treatment (17), and those who do receive treatment are treated primarily by general practitioners (18). Integration of mental health treatment into primary care increases access (19), decreases stigma (20), has positive outcomes (21), and appears to be cost-effective (22).

## Integration of Primary Care Into Mental Health Care

Medical illnesses are prevalent among people who have serious mental illness (23), yet medical illness is often untreated or poorly treated in this population (24). Integration increases access to primary care and improves health outcomes. A randomized trial of an integrated model of primary care for people with serious mental illness found that people who received integrated care were more likely to have had a primary care visit, had more primary care visits, were more likely to receive preventive care, and had a greater improvement in health than people who received routine medical care (25).

Failure to provide integrated treatment leads to undertreatment of mental illness and of medical illness among people with serious mental illness. Untreated mental illness is costly because it contributes to disability and higher overall health care use (26), and untreated and undertreated medical illness among people with serious mental illness contributes to accelerated mortality (27).

## Children and Adolescents: Starting at the Beginning

Psychiatric symptoms and persistent mental illness often manifest when a person is young and require early intervention (28,29). Fifty percent of all mental illnesses will emerge by the time a person is 14 years old; 75% will be present by age 24 (30). These are primarily anxiety and mood disorders, attention deficit-hyperactivity disorder (ADHD), eating disorders, and psychotic illnesses. Childhood mental illnesses often begin as less serious illnesses and are highly treatable, but multiyear lags in entering care are common (31). Mental illness produces great distress in children, is a barrier to educational performance, and adds to family tensions and discord.

Mental illness is also among the principal reasons children appear in doctors' offices — though seldom with the complaint of a mental illness (28). For many reasons (eg, lack of training and familiarity, inadequate reimbursement of clinician time to conduct thorough assessments) pediatricians in settings without mental health staff, training, or consultative support often fail to detect mental illnesses (28). Relying on specialty providers to address pediatric mental illness will not work; the supply of child psychiatrists is a fraction of the need, and the gap is worsening (32). The lack of pediatric mental health services contributes to long delays in entering care (17) and to less serious problems (eg, mild adjustment problems, mild depression or anxiety) becoming more serious conditions (eg, conduct disorder, major depressive disorder) years later. For serious mental illnesses, including psychotic and severe mood disorders, studies have identified degenerative changes in brain structure and functioning (especially in the frontal lobes) early in the course of illness, indicating that delay in detection and treatment is neurotoxic for these people and associated with poor prognosis (33).

The American Academy of Pediatrics (AAP) has recognized the prevalence of child mental illnesses, their relationship to adverse early childhood experiences (34), the undersupply of mental health specialists, and that pediatric practices can — with the right staffing and supports — provide excellent care for many children with mental illnesses. Moreover, early intervention works (35). In 2006, AAP released a pediatric tool kit titled Feelings Need Check Ups Too (www.aap.org/disasters/pdf/Feelings%20Need%20Check%20ups%20Toolkit_0823.pdf), including diagnostic tools, treatment algorithms, and other resources. AAP also released a comprehensive report outlining mental health competencies for pediatric primary care (36). In conjunction with the American Academy of Child and Adolescent Psychiatry, AAP proposed steps to reduce administrative and financial barriers to collaboration between primary care and mental health services (37). These national reports follow recommendations of the US Preventive Services Task Force regarding depression screening in adolescents, which reported that such screening is feasible and indicated because effective interventions for adolescent depression are available (38).

## Mental Illness Among Adults in General Medical Care Settings

As with children, most adults with a mental illness are seen in primary care, not by mental health specialists. Among adults who received care for a past-year episode of major depressive disorder, approximately 60% saw a general practitioner or family doctor, whereas approximately 30% saw a psychiatrist or psychotherapist (39). Although primary care is the major locus of treatment for mental illness, many patients in primary care are never diagnosed or treated.

Modest improvements in primary care mental health practices increase detection and evidence-based treatment of common mental illnesses for adults (ie, depression, anxiety disorders, and problem drinking) (40,41). The recognition and treatment of depression were boosted considerably by the introduction and aggressive marketing of selective serotonin reuptake inhibitors (SSRIs) since the late 1980s. Compared with previous treatments (eg, tricyclic antidepressants), SSRIs have fewer side effects, greater safety, and easier dosing. In the United States, SSRI prescribing for all conditions doubled from 1996 through 2006 (40), indicating that more patients were receiving treatment. Similarly, brief, simple primary care interventions for patients with problem drinking are effective (41).

Despite these improvements in treating common mental illnesses, improving mental health care in primary care remains challenging. Many patients treated for depression in primary care receive an inadequate trial of medication (insufficient dosage or duration of treatment or both) (42). In addition, the percentage of patients receiving treatments likely to be effective has declined since 2004, when black box warnings were first included on antidepressant labels (42).

We now have robust evidence of what it takes to improve treatment of depression — and by extension other common mental illnesses — in primary care settings (22). Interventions described as *collaborative care* include embedding mental health professionals (such as social workers or nurses) in the primary care setting with accessible psychiatric consultation, screening for mental illnesses, establishing clear treatment guidelines, and measuring the patient's condition periodically. Collaborative care roughly doubles positive depression treatment outcomes; in one study, 45% of patients randomly assigned to a collaborative care intervention had a 50% or higher reduction in depressive symptoms from baseline compared with 19% of usual care participants (22).

## Adults With Serious Mental Illnesses

In the public mental health system, which includes public psychiatric hospitals, community mental health clinics, rehabilitation programs, and supportive housing, integrated medical care is often absent. Adults with serious mental illness who receive care in the public mental health sector die on average 25 years earlier than people in the general population (43). Many people with serious mental illness smoke heavily (3 of 4 are nicotine-dependent), eat poorly, are sedentary, and lack preventive and ongoing physical health care (44). To make matters worse, some of the leading medications for psychotic illness increase risk for weight gain, diabetes, and cardiovascular disease (45). Integrating medical care in mental health specialty venues makes sense because these people, as a rule, have their medical home as the mental health clinic — not the primary care clinic (13).

## Common Elements of Integration

The common ground for integration is the adoption of, as standards of care, elements we identified for both primary care and mental health settings. These standards include screening for co-occurring mental illnesses in primary care settings and for co-occurring health problems in mental health settings. Clear, feasible clinical care paths should be adopted for treating common conditions and for referring to specialty care (eg, because of complexity, safety, lack of response). Agencies should embed or collocate mental health staff in primary care settings to assist with screening, counseling, and care monitoring and coordination. Likewise, primary care staff should be collocated in mental health agencies to treat or manage low-complexity health problems and coordinate care for complex cases.

Provider agencies need to create readily accessible (eg, by telephone within minutes) consultation in which psychiatrists are available to pediatricians, obstetricians and gynecologists, and primary care physicians. They must also continually measure parameters of health or mental health functioning by using meaningful and practical measures of blood pressure, body mass index, smoking status, and depression. Finally, agencies should use a single clinical record (preferably electronic, with decision support, prompts, and ongoing clinical performance monitoring) except when specialty care considerations require a distinct record.

## From Isolated Demonstrations to Everyday Practice

Whereas the research projects on collaborative care are well known, there are also many home-grown examples of what can be done. Several projects are under way through the New York State Office of Mental Health (NYS OMH) and the New York City Department of Health and Mental Hygiene (NYC DOHMH), and these agencies are not unique in their efforts.

In early 2009, all 66 NYS OMH-operated mental health clinics, which had 15,000 adult outpatients, began systematically collecting 3 health indicators: blood pressure, body mass index, and smoking status. Within months, information had been collected on approximately 50% of adult outpatients, with a goal of 100% within a year (Sheila Donahue, NYS OMH, oral communication, December 2009). Collecting this information is premised on the belief that what gets measured gets managed, and is meant to promote the expansion of wellness programs and primary care collaborations in these mental health clinics.

Various NYS OMH and nonprofit community mental health agencies have established medical clinics on-site at their mental health centers. Some are collocated, though remaining separate entities; others are operated by the mental health clinic. The New York State Department of Health has funded 6 demonstration projects that identify high-need people with physical and mental illnesses whose integrated care will be the responsibility of accountable mental health agencies (46).

In 2005, the NYC DOHMH began a citywide initiative to implement depression screening and specified care management in 100% of New York City's primary care practices. This work continues and is reported elsewhere (47,48).

## Conclusions

The medical home concept is a centerpiece of health care reform in this country. The goals for a medical home are that it be accessible, comprehensive, coordinated, culturally responsive, person-centered, and compassionate. It would be an accountable entity where patients and families feel that their interests are primary and attended to by caring clinicians. These goals cannot be achieved if there is no room for mental health in the medical home.

"There is no health without mental health," said Dr David Satcher in his first Surgeon General's report on mental health (28). The integration of health and mental health is not only possible, it is essential to the success of health reform. Integration is critical to moving away from episodic acute care to prevention, wellness, and primary care. Leaving mental health out of health care produces greater suffering for both health and mental health conditions, greater burden to families and communities, and far greater health expenditures.
