# Erratum, Vol. 7: Issue 6

**Published:** 2010-12-15

In the article "Making Room for Mental Health in the Medical Home," the degree for Ilana R. Nossel, MD, was omitted. The correction was made to our website on October 26, 2010, and appears online at http://www.cdc.gov/pcd/issues/2010/nov/09_0198.htm. We regret any confusion or inconvenience this error may have caused.

En el artículo "Integración de la salud mental en el concepto de "hogar médico", se omitió la mención del título académico de la Dra. Ilana R. Nossel. La corrección se hizo en nuestro sitio web el 26 de octubre del 2010, y se puede encontrar en línea en http://www.cdc.gov/pcd/issues/2010/nov/09_0198_es.htm. Lamentamos cualquier inconveniente o confusión que este error haya podido causar.

